# Spaceborne Satellite for Snow Cover and Hydrological Characteristic of the Gilgit River Basin, Hindukush–Karakoram Mountains, Pakistan

**DOI:** 10.3390/s19030531

**Published:** 2019-01-27

**Authors:** Dostdar Hussain, Chung-Yen Kuo, Abdul Hameed, Kuo-Hsin Tseng, Bulbul Jan, Nasir Abbas, Huan-Chin Kao, Wen-Hau Lan, Moslem Imani

**Affiliations:** 1 Department of Geomatics, National Cheng Kung University, No. 1,University Road, Tainan City 70101, Taiwan; kuo70@mail.ncku.edu.tw (C.-Y.K.); p68031018@mail.ncku.edu.tw (H.-C.K.); p68001021@mail.ncku.edu.tw (W.-H.L.); mimani@mail.ncku.edu.tw (M.I.); 2 Integrated Mountain Area Research Centre (IMARC), Karakoram International University (KIU), Gilgit 15100, Pakistan; 3 Government Degree College Danyore, Gilgit 151100, Pakistan; abdul_hameed733@yahoo.com; 4 Center for Space and Remote Sensing Research, National Central University, Taoyuan City 32001, Taiwan; khtseng@csrsr.ncu.edu.tw; 5 Institute of Hydrological and Oceanic Sciences, National Central University, Taoyuan City 32001, Taiwan; 6 Institute of Space and Planetary Astrophysics, University of Karachi, Karachi 75840, Pakistan; bulbul.gilgit@gmail.com; 7 Department of Geography, Government College University Lahore, Lahore 54000, Pakistan; nasir_gis_geo@hotmail.com

**Keywords:** Gilgit Basin, moderate resolution imaging spectroradiometer (MODIS), snow-covered area, hydrological regime

## Abstract

The Indus River, which flows through China, India, and Pakistan, is mainly fed by melting snow and glaciers that are spread across the Hindukush–Karakoram–Himalaya Mountains. The downstream population of the Indus Plain heavily relies on this water resource for drinking, irrigation, and hydropower generation. Therefore, its river runoff variability must be properly monitored. Gilgit Basin, the northwestern part of the Upper Indus Basin, is selected for studying cryosphere dynamics and its implications on river runoff. In this study, 8-day snow products (MOD10A2) of moderate resolution imaging spectroradiometer, from 2001 to 2015 are selected to access the snow-covered area (SCA) in the catchment. A non-parametric Mann–Kendall test and Sen’s slope are calculated to assess whether a significant trend exists in the SCA time series data. Then, data from ground observatories for 1995–2013 are analyzed to demonstrate annual and seasonal signals in air temperature and precipitation. Results indicate that the annual and seasonal mean of SCA show a non-significant decreasing trend, but the autumn season shows a statistically significant decreasing SCA with a slope of −198.36 km^2^/year. The annual mean temperature and precipitation show an increasing trend with highest values of slope 0.05 °C/year and 14.98 mm/year, respectively. Furthermore, Pearson correlation coefficients are calculated for the hydro-meteorological data to demonstrate any possible relationship. The SCA is affirmed to have a highly negative correlation with mean temperature and runoff. Meanwhile, SCA has a very weak relation with precipitation data. The Pearson correlation coefficient between SCA and runoff is −0.82, which confirms that the Gilgit River runoff largely depends on the melting of snow cover rather than direct precipitation. The study indicates that the SCA slightly decreased for the study period, which depicts a possible impact of global warming on this mountainous region.

## 1. Introduction 

The Asian high mountain ranges of Himalaya, Karakoram, and Hindukush (HKH) are collectively termed the “third pole of our planet” [1]. These mountains, which provide freshwater to 1.3 billion people in the Indo–Gangatic plains, are also called the “Water Towers of Asia” [2]. This region contains many alpine glaciers that provide fresh water for agriculture, industry, and domestic usage. The Indus Basin, which originates from the Tibetan plateau, is the one of the largest river basins in Asia. The river subsequently runs through northern areas of Pakistan and finally falls into the Arabian Sea. Snow and ice melts, the main sources of the Indus River, play irreplaceable roles in Pakistan’s economy [3]. The Indus River flow is stored in the Tarbela reservoir, providing water flow to the agriculture land of Pakistan through the Indus irrigation system. The upstream area of the Tarbela reservoir is called the Upper Indus Basin (UIB). Almost 11.5% of the total UIB area is composed of perennial glacial ice dominated by large valley glaciers [4]. The Greater Karakoram Range in UIB, which covers an area of 16,300 km^2^, has a vast area of glaciers owing to high altitude [5,6]. Hewitt K. [7] reported that 90% of total glaciated area in Karakorum Range lies between 5000–6000 m, which represents the accumulation zone of the glaciers. 

Snow is a vital resource in mountainous regions, not only for water supply and hydropower generation, but also for the environmental aspects of the local mountain flora and fauna [8]. In the climate change context, the impact of snow cover changes on hydrologic response is of great interest for many cryosphere scientists. Various climatic factors, such as precipitation, temperature, wind speed, and solar radiation, affect snow and glacier growth and movement. Variations in glacier mass and hydrological cycle are linked with over several decades of global warming [9]. According to the IPCC statement [10], the air temperature increased by 0.6 °C globally in late 19th century, and it is predicted to rise by 1.5–5.8 °C over the next hundred years. This increase in air temperature has reduced the snow-covered area (SCA) by 10% since the late 1960s, and it is predicted to further reduce during the 21st century according to the Food and Agriculture Organization of the United Nations.

For the investigation of basin-scale phenomena, satellite remote sensing methods have been widely used for cryosphere dynamics in mountainous regions across the world. It has been validated that, the snow product of moderate resolution imaging spectroradiometer (MODIS) could be used in high-altitude areas for determining snow variations and snowmelt runoff modelling [11,12,13]. An assessment of MODIS snow coverage with ground base observations performed by Takeli et al. [14] found that the estimated snow cover is accurate even in a river basin with rugged terrain. Lee et al. [13] predicted stream flow with significant accuracy by using MODIS snow product in the snow melt runoff model in the Upper Rio Grande Basin, which is snowmelt dominated. Immerzeel et al. [11] studied the SCA influence on river runoff in the UIB and found that the runoff can be precisely predicted by using MODIS snow cover as an input data to the hydrological runoff model.

Contrary to the other cryosphere regions of the world, several investigations have asserted that the UIB is a region with an increased tendency for cryosphere areas, probably due to the decreasing trend in summer temperature and increasing winter precipitation [7,15,16]. Hewitt [7] reported that a majority of glaciers are surging in the Central Karakoram Range. Seasonal trends of summer mean air temperature (July–September) which is the key for glacier melt, showed a decreasing trend at several climate stations in the Karakoram region during 1961–2000 [17]. A similar temperature decline trend was reported by Hussain et al. [18] in monsoon and pre-monsoon periods for that region. Tahir et al. [4] studied the remote sensing snow data and found that the decreased flow trend in the Hunza River was caused by a decreasing trend in summer mean temperatures and increasing trend in winter precipitation. The authors concluded that cryosphere has a slight expansion in the Hunza River Basin. However, studies on the snowfall amount in the region of Gilgit Basin over the past few years and how this inconsistency might affect the water scheme of the UIB most important rivers, which have been under consideration for several hydroelectric projects [19], are lacking. Similarly, this variation might affect regional water management for different purposes such as drinking, irrigation, and hydropower generation, consequently leading to unsustainable water use conditions under the future scenarios of lower resource availability. 

Effective management of water resources for sustainable long-term economic growth and rectifying widespread food insecurity and nutrition deficiencies are very important for the country. Therefore, the main objective of this research is to investigate the spatial and temporal variability of cryosphere dynamics in the Gilgit River Basin based on the satellite images and in-situ data of hydro-meteorology.

## 2. Materials and Methods

### 2.1. Study Area

**Figure 1:**
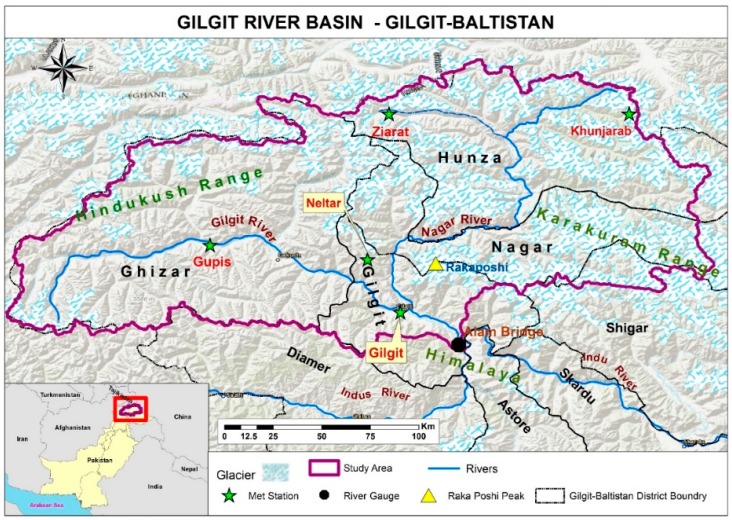
Study area in northern Pakistan.

**Figure 2:**
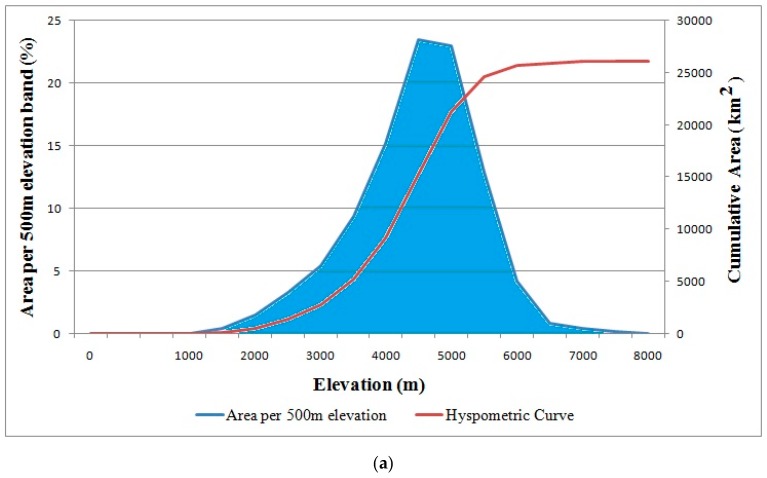

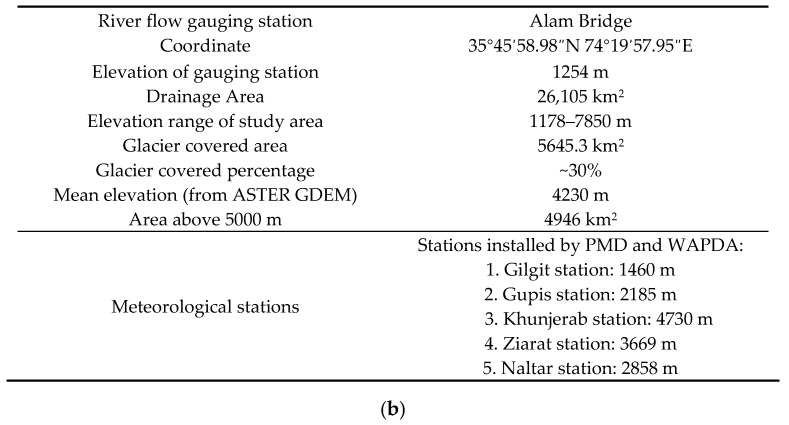
(**a**) Hypsometric curve of Gilgit River basin and distribution of the area by elevation. (**b**) Gilgit Basin key characteristics.

Figure 1 shows that our study area covers the Gilgit Basin (36.37° N, 74.19° E at the center), which is a part of UIB in the mountain chains of Karakoram and Hindukush ranges. The area comprises the Gilgit, Hunza, Nagar, and Ghizer districts of the Gilgit–Baltistan Province. The basin comprises valleys, steep mountains, and a few of the highest peaks of the Great Karakoram Ranges. It ranges from 1178–7850 m, where 88% of the total area lies between 4000–5500 m (Figure 2a). Figure 2b shows the key characteristics of geographical settings. The drainage area of the basin calculated at gauging station is about 26,105 km^2^ with the mean elevation of about 4230 m. About 4946 km^2^ area lies above 5000 m elevation is mainly covered by snow and glaciers. The glaciers and seasonal snow melt significantly contributes to the river runoff of the basin. The maximum snow cover area approximately 70–80% in winter and decreases to 20–30% in summer. Any change in climate will impact on the frozen water resources of the basin which eventually affect the river runoff. According to the data record of WAPDA from 1961–2010, the Gilgit River mean annual discharge at the gauging station about 288.63 m^3^/s. 

The annual precipitation in the UIB that occurs in the winter and spring originates from the western disturbances [6]. Various meteorological stations have been installed from east to west. Gilgit basin includes five climate stations: Gilgit, Gupis, Khunjerab, Ziarate and Naltar. The mean total annual precipitation is 132 mm at Gilgit (1460 m), 314 mm in Gupis (2185 m), 170 mm in Khunjerab (4730 m), 22 mm in Ziarat (3669 m) and 680 mm in Naltar (2858 m) according to the record from 1999–2008. Climatic indicators are very strongly influenced by altitude because the area comprises steep mountains that produce sharp ecological zones. Valley floors are arid with merely 100–200 mm annual precipitation [7]. The area above 3500 m receives maximum precipitation in Karakoram Range [7]. According to the same study, over the elevation of 5000 m, a significant drop of temperature and five to tenfold increase in precipitation were observed. By combining the snow cover and ablation models, Winiger et al. [20] calculated annual precipitation for different altitudinal zones. More than 1700 mm/year total annual precipitation was calculated for the northwest Karakoram over 5500 m.

### 2.2. MODIS Snow Cover 

**Figure 3:**
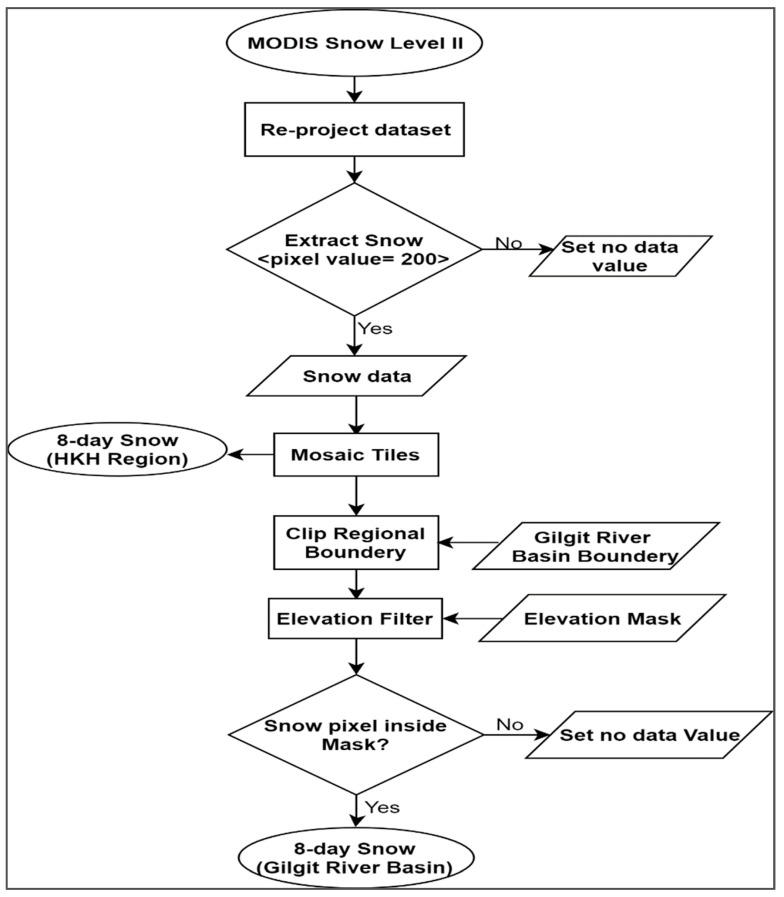
Process for snow extraction.

The MODIS is a global remote sensing satellite that provides imageries of the earth surface in 36 spectral bands of the wavelength between 0.405 to 14.385 µm. MODIS/Terra L3 8-day composite snow cover data (MOD10A2) are provided with a sinusoidal map projection of a 500-meter resolution. MODIS filters cloud images and preserves the maximum snow cover extent over an 8-day period in compressed hierarchical data format, along with corresponding metadata. However, the cloud cover and misclassifications of snow are the major restrictions for the hydrological studies of MODIS snow data. Snow and cloud confusion leads to overestimation errors [21,22] and these errors would be further propagate into 8-day snow product due to incorrect identification of snow in MODIS daily products [23]. The over estimation of snow can be reduce by applying several algorithms developed by [21,24,25]. In our study, we did not calculate overestimation errors in 8-day snow product that’s beyond the scope of this study. However, the minimum cloud cover (<20%) datasets of 694 MOD10A2-V05 images from 1 January 2001 to 27 December 2015 was acquired from the NASA National Snow and Ice Data Centre (http://nsidc.org/data/MOD10A2). Furthermore, daily snow data product (MOD10A1/MYD10A1) was not used due to severer cloud contamination in daily snow maps. For every 8-day image, two tiles have to be mosaicked to cover the study area. These images were re-projected into geographic coordinate system (GCS)-WGS 1984, and snow pixel values of the MODIS images were extracted. The snow cover corresponding to snow pixel on image was multiplied by a 0.25 km^2^ to convert it into area. This area is represented as SCA. The main processes for snow extraction are shown in Figure 3.

To investigate the snow cover discrepancy, monthly anomalies were calculated on SCA data by using the technique mentioned in [26]:

(1) $$xa_{i}=\left(x_{i}-\bar{x}\right)$$

where $xa_{i}$ is the SCA anomaly value, $x_{i}$ is the annual or seasonal SCA, and the $\bar{x}$ is the mean value for the annual or seasonal period for the years in the time series. The estimated SCA anomalies by using the above equation were used to determine the trend in which the slope (i.e., linear rate of change) was calculated by using the Sen’s slope estimator (Equation 2). The Sen’s slope is calculated as the median ($d_{k}$) of all slopes:

(2) $$d_{k}=\frac{y_{j}-y_{i}}{x_{j}-x_{i}}\;\;\;\;\;\;i<j$$

where $d_{k}$ is the slope series among the all combinations of ordered pairs, which are numbers from 1 to k:

(3) $$k=\frac{\left(n\right)\left(n-1\right)}{2}$$

where n is the total number of ordered pairs. To estimate the trend in time series data, Mann–Kendall Test (MKT) was performed. The non-parametric MKT is a robust technique widely used to determine trends in environmental variable [27]. The MKT statistics on the time series data is calculated according to:

(4) $$t=\overset{n_{-1}}{\underset{i=1}{\sum }}\;\overset{n}{\underset{j=i+1}{\sum }}Signo\left(x_{j}-x_{i}\right)$$

The positive value of t shows an increasing trend and vice versa. The signo(x) can be calculated as:

(5) $$signo\left(x\right)=\{\begin{array}{c} +1\;\;\;\;\;x>0\\ 0\;\;\;\;\;\;x=0\\ -1\;\;\;\;\;x<0 \end{array}$$

### 2.3. Topographic Reference

The advanced spaceborne thermal emission and reflection radiometer (ASTER) global digital elevation model (GDEM) (Ministry of Economy Trade and Industry and NASA) contains topographic information of global terrain resolution at 30 m. It is used to delineate between the watershed and further topographic studies. The Ministry of Economy Trade and Industry of Japan and NASA (USA) jointly launched an ASTER sensor onboard Terra that has been offering visible near infrared, shortwave infrared, and thermal infrared global data since June 2009. Nineteen ASTER GDEM2 tiles were mosaicked and further processed to delineate the Gilgit Basin by using the Hydrology tool in ArcGIS (Environmental Systems Research Institute (ESRI), ver. 10.3). The total basin area from Alam Bridge (considered as pour point) that was calculated for this study is 26,105 km^2^. This calculation is nearly similar to that provided by the Water and Power Development Authority (26,159 km^2^) in a surface water hydrology project [3]. In addition, an area of 27,525 km^2^ was given by Archer D. [16]. A<5% difference in area calculation is mainly caused by auto delineation where iterative pit-filling can generate spurious drainage networks and basins, although internal drainage areas are adjacent [28]. Subsequently, the extracted area was superimposed by hydro-meteorological stations, administrative boundaries, glacier, and other relevant layers (Figure 1).

### 2.4. SCA and In-Situ Data

The in-situ observations of stream flow from 2001–2013 and climate stations data from 1995–2013 with in the basin were used in this study. Hydro-meteorological mean monthly data were averaged to obtain a representative value at the basin level. For air temperature and precipitation, the annual average and annual cumulative were calculated as:

(6) $$\bar{T}=\frac{\sum_{i=1}^{n}T_{i}}{n},\;\;\;\;\;\;\;\;\;\;\;\;\;\;\;\;\;\;\bar{P}=\frac{\sum_{i=1}^{n}P_{i}}{n}$$

where $\bar{T}$ is the annual mean temperature, n is the number of stations in the basin, $T_{i}$ is the air temperature measured by specific station, $\bar{P}$ is the annual accumulated precipitation in the basin, and $P_{i}$ is total annual precipitation measured by one specific weather station. For the stream flow, the mean monthly runoff measured by a gauging station was used.

Pearson’s correlation technique was used to analyze the relationship between MODIS-derived snow coverage, stream flow, temperature, and precipitation data. Relationship between climate data and annual/seasonal stream flow was determined and significant results are tested with help of p-values. Regression analysis between SCA, climate data, and stream flow data were also determined by using the XLSTAT software extension in MS Excel.

Linear regression technique depicts the calculation of dependent variable (Snow cover) from a known value of independent variable (climatic variables) and stream flow. Therefore, it is the measurement of average relationship between two or more variables based on the original unit of data. A model of multiple regression between a response variable (snow cover) and an explanatory variable (climatic variables) and stream flow) is expected. 

## 3. Results 

### 3.1. Snow Cover Dynamics

**Figure 4:**
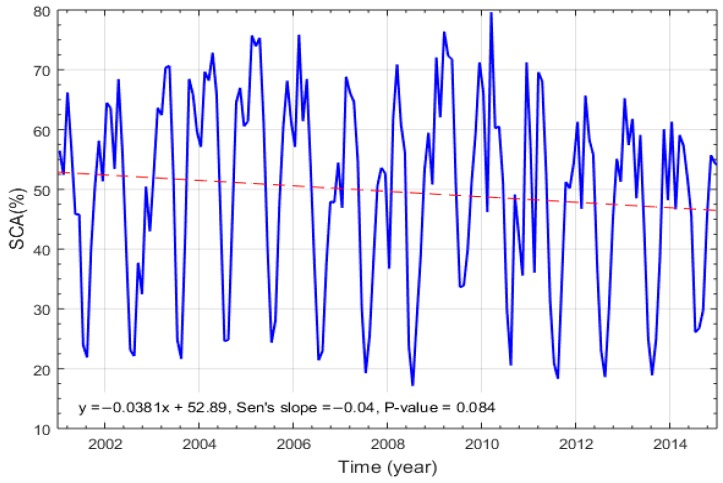
Snow cover trend in Gilgit River Basin from 2001 to 2015 by using MODIS 8-day product.

The snow cover variability for the period 2001–2015 in the Gilgit River Basin is shown in Figure 4. The x-axis shows the time period while the y-axis indicates snow cover area in percentage (%). The red dotted line shows the trend while the blue solid line shows SCA (%) variability. Sen’s Slope is used to determine the rate of observed changes and the P-value decide whether there is statistically significant trend in the data set. A decreasing trend is observed with the slope of −0.04 km^2^/year, although the trend is not statistically significant (p<0.05). However, a sharp decline trend observed from 2010–2015.

**Figure 5:**
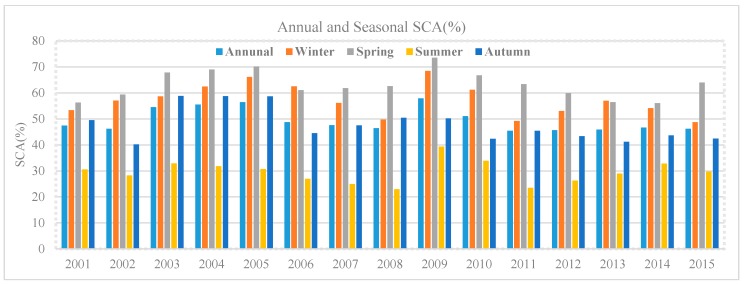
Annual and Seasonal mean SCA (%) for the period 2001–2015.

**Figure 6:**
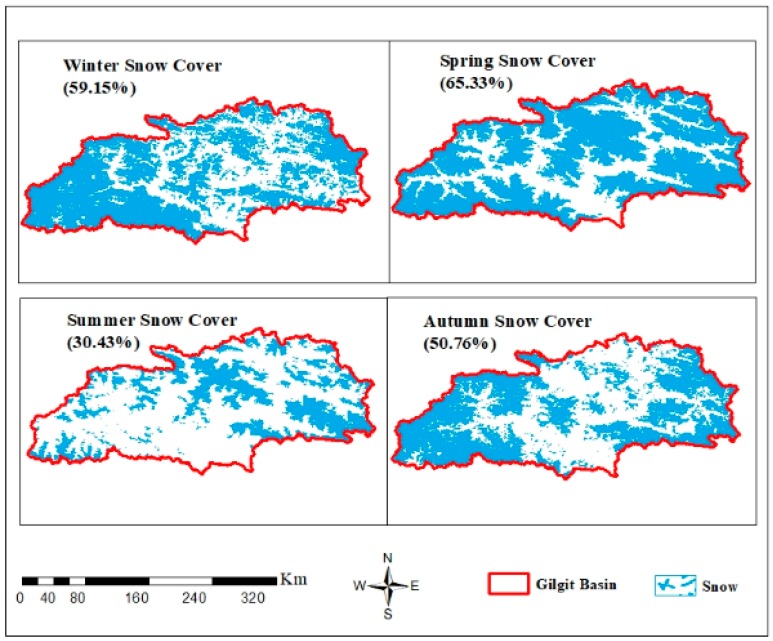
Average seasonal SCA in the Gilgit River Basin during 2001–2015 for winter (DJF), spring (MAM), summer (JJA), and autumn (SON).

The annual and seasonal mean SCAs (%) for 2001–2015 are shown in Figure 5. The maximum annual SCA found in the years 2009 and 2005 were 57.86 % and 56.44% in the catchment area, respectively. Spring and winter seasons indicate the highest accumulation of SCA. The maximum mean SCA in autumn occurred in 2003, 2004 and 2005. The maximum mean SCA in summer occurred in 2009. The minimum annual mean SCA occurred in 2011 and 2012. The seasonal average SCAs (%) for the study period are shown in Figure 6. Gilgit River Basin boundary is shown in red while the blue color indicates the snow cover. The average SCA for winter season (59.15 %), spring (65.33 %), summer (30.43 %), and autumn (50.76 %). Spring season has the greatest SCA compared with winter season, which might mean more snowfall occurs in spring instead of winter, while summer has the minimum SCA. 

**Figure 7:**
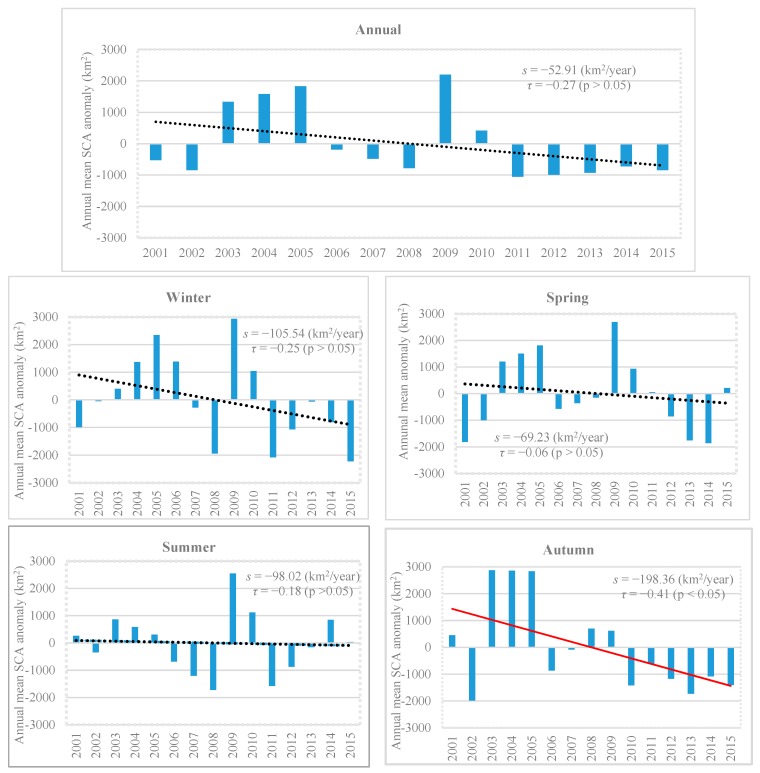
Annual and seasonal mean SCA anomalies for the period 2001–2015. Mann Kendall’s trend test “τ” and Sens’s slope estimator “s”.

The annual and seasonal SCA anomalies for 2001–2015 are shown in Figure 7. Annually, 2009 is 2198.03 km^2^ above the mean for the study period, whereas 2011 and 2012 are 1053.68 and 990.98 km^2^, respectively, below the average for the period. In addition, negative anomalies are observed during 2011–2015, whereas positive anomalies are observed nearly 1333 km^2^ above the mean during 2003–2005. The year 2009 shows the maximum positive anomalies in the winter and spring seasons, whereas the maximum negative anomaly occurs in 2015 with 2216.83 km^2^ in winter and in spring of 2014 with 1859.23 km^2^. In summer, the maximum positive anomaly occurs in 2009 with 2548.88 km^2^, whereas the maximum negative anomaly occurs in 2008 with 1724.41 km^2^. The period 2003–2005 presents the maximum positive anomaly nearly 2874 km^2^ above the mean for the autumn season, whereas 2002 presents maximum negative anomaly of 1979.39 km^2^. However, negative anomalies are observed during 2010–2015 for the same season.

A decreasing trend is observed for the annual mean SCA at the rate of −52.91 km^2^/year, but the trend is not significant statistically as shown in Figure 7. The winter and spring seasons show non-significant decreasing rates of −105.54 and −69.23 km^2^/year, respectively. Likewise, the summer season shows a decreasing rate with a slope of −98.02 km^2^/year; however, the autumn season shows a decreasing trend, which is significant (p<0.05) at the rate of −198.36 km^2^/year. Furthermore, in autumn starting in 2010, the annual SCA indicates a negative trend of about 608 km^2^/year below the average for the period of 2001–2015. 

### 3.2. Climatic Variability Analysis

To assess the correlation between climate variables (monthly temperature and precipitation) recorded by different climate stations in the basin, a Pearson’s correlation test (significance level, p=0.05) is used as shown in Table 1. A highly strong correlation is shown by all climate stations in the basin for maximum, minimum, and mean temperature with significant p-value < 0.05. The correlation coefficient value is at a minimum of 0.816 in each case. The monthly mean temperature (Tmean) has a maximum correlation coefficient (r = 0.986) between Khunjerab and Ziarat and the minimum (r = 0.945) between Khunjerab and Naltar. Cumulative monthly precipitation also indicates a significant correlation among the Gilgit River climate stations. The highest correlation coefficient is calculated between Gilgit and Gupis (r = 0.624) while the statistically significant minimum correlation coefficient was found between Gupis and Khunjerab (r = 0.139), followed by Gilgit and Khunjerab (r = 0.168).

The non-parametric test, MKT, is used to analyze whether a monotonic increasing or decreasing trend of the climate variables is observed during 1995–2013 as shown in Table 2. The analysis results indicate that annual mean temperature in most of the stations shows an upward trend except for the Naltar station, which indicates non-significant negative trend that is −0.06 °C/year. The highest increasing rate of annual temperature with a slope of 0.05 °C/year is at Khunjerab station followed by Ziarat with a slope of 0.03 °C/year. Gilgit and Gupis stations also shows and increasing trend with the same slope of 0.02 °C/year. The summer temperature indicates an increasing trend in majority of the stations except Ziarat and Naltar which shows non-significant negative trend with slope of −0.01 and −0.09 °C/year respectively. Similarly, the increasing trend of summer temperature in Gilgit (0.02 °C/year) and Gupis stations is 0.02 °C/year. Winter temperature also shows an upward trend except the Naltar station. The two highest elevation stations, Khunjerab and Ziarat indicates highest increasing rate of winter temperature with a slope of 0.17 and 0.01 °C/year, respectively. Furthermore, Giligt has an increasing trend of 0.05 °C/year and the Gupis station, 0.01 °C/year.

The precipitation record of climate stations in Gilgit River Basin indicates an increasing trend in all stations except for Gilgit and Gupis which shows a non-significant decreasing trend with the slope of −1.9 and –9.56 mm/year. The highest increasing rate of precipitation is observed in Naltar station with an increasing slope of 14.98 mm/year. Khunjerab and Ziarat also show an increasing trend with a slope of 2.75 and 7.3 mm/year, respectively.

### 3.3. Correlation Between Snow Dynamics, Climate Variables, and Stream Flow in the Gilgit River Basin

**Figure 8:**
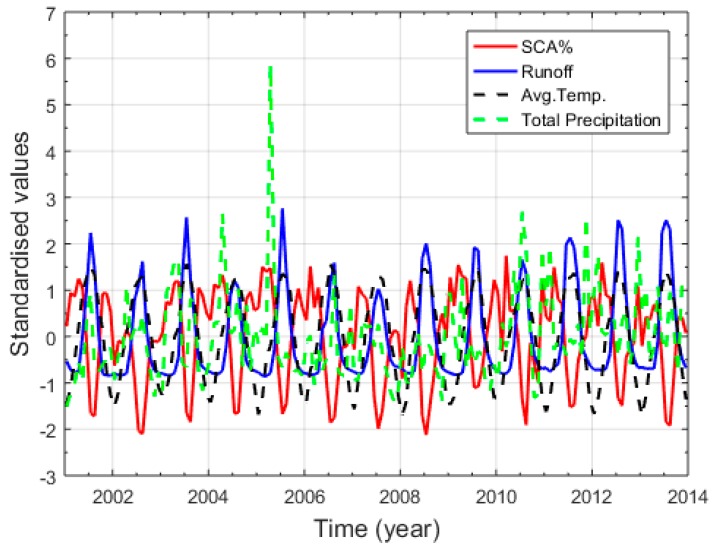
Standardized values of runoff, SCA, precipitation, and average temperature.

**Figure 9:**
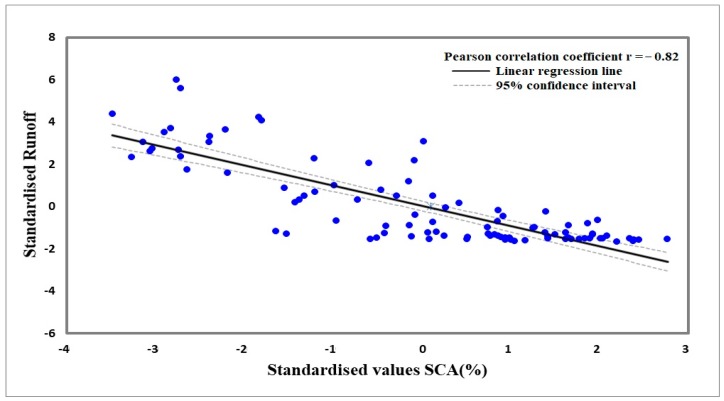
Correlation between standardized values of runoff and SCA.

The SCA is at its maximum in winter and spring and at its minimum in summer (Figure 6); such a variation in SCA has a negative correlation with mean temperature and river runoff, whereas it has a positive correlation with the precipitation as shown in Table 3. To determine the relationship among the variables (snow cover, precipitation, temperature, and runoff), standardized values are calculated by dividing the standard deviation of the distribution by the difference of each data point from the mean. The standardized value relationship of the variables is presented in Figure 8. SCA has a negative correlation with mean temperature and stream flow in the Gilgit River, showing that the SCA decreases in summer with the increase of mean temperature in the basin, subsequently increasing river runoff. By contrast, the SCA increases with the decrease of temperature in winter, thereby resulting in decreased river runoff. SCA has a direct relationship with the precipitation data, but the correlation is not significant. The precipitation value is at a maximum in April and May 2005 because the entire basin receives high precipitation during these two months (Figure 8). (Gupis Station: 344 mm, Gilgit: 58 mm, Naltar: 192 mm, Ziarat: 47 mm, and Khunjerab: 17 mm). Figure 9 shows the high correlation coefficient of −0.82 between the standardized values of SCA and Gilgit River flow, which represents that the variation in river flow highly depends on the SCA change in the basin. For the understanding of the annual and seasonal variations among SCA, stream flow and climate variables, the Pearson correlation coefficient is applied. Table 3 describes the annual and seasonal coefficient values for the Gilgit River basin (2001–2013). 

The Pearson correlation is considered to estimate dependencies among the climate variables (minimum, maximum, and mean temperatures; precipitation), snow cover dynamics and stream flow in the Gilgit River at Alam Bridge. A highly significant negative correlation is found between SCA and mean annual temperatures as observed at Gilgit (r = −0.612), Khunjerab (r = −0.662), Gupis (r = −0.626), Naltar (r = −0.653), and Ziarat (r = −0.683). Meanwhile, the average of the entire Gilgit Basin is found at r = −0.652. A strongly significant negative correlation is found between summer SCA and temperatures in the Gilgit Basin while winter temperature has a negative correlation with the winter SCA, which is not statistically significant (Table 3). The SCA has a significantly negative correlation with stream flow in the catchment. Snow coverage has a correlation coefficient of −0.806 compared with the mean annual runoff. For summer stream flow, a value of −0.836 shows that the Gilgit River stream flow highly depends on the SCA and mean temperatures. 

Furthermore, a weak correlation was found between the SCA and precipitation in Gilgit (r = 0.001), Gupis (r = 0.093), Khunjerab (r = −0.124), Ziarat (r = 0.121), and Naltar (r = 0.060), or in the entire catchment (r = 0.076). Summer SCA has a significant positive correlation with the precipitation except the Khunjerab station which has significant negative correlation coefficient of −0.274 while Ziarat station shows statistically non-significant value of r =0.079. Winter SCA has a non-significant positive correlation with precipitation however, the Gupis station shows a positive statistically significant (p<0.05) correlation value of r = 0.233 as shown in Table 3. Summer stream flow has a positive correlation (r = 0.063) with winter SCA, however the correlation is not statistically significant.

### 3.4. Hydrological Behavior of the Gilgit River Basin

The Pearson correlation statistics was used to explore the hydrological behavior of the Gilgit Basin. Table 4 provides the correlation between annual and seasonal stream flows at Alam Bridge and climate data from the Gilgit Basin climate stations. The impact of temperature on the flow magnitude of Gilgit River can be determined through the correlation coefficients between these two variables. For annual temperature (maximum, minimum, and mean) and runoff at Alam Bridge, the Gilgit Basin climate stations show a significantly high correlation. 

A significant positive correlation is found between runoff at Alam Bridge and annual mean temperature at Gilgit (r = 0.815), Khunjerab (r = 0.836), Gupis (r = 0.810), Naltar (r = 0.784), and Ziarat (r = 0.846) with considered p-values < 0.05. A highly positive correlation exists between stream flow and summer temperature: the highest significant correlation (r = 0.914) is shown by Gilgit climate station followed by Ziarat (r = 0.909) and Naltat (r = 0.750). The winter mean temperature also shows significant positive correlation with the runoff, and the coefficient value is not less than 0.459 for all stations.Summer runoff is said to be certainly dependent on winter precipitation of the UIB [17]. Correlation values show insignificant positive trends for Gilgit (0.189) and Gupis (r = 0.139) because p-values > 0.05, while some stations shown week positive correlation i.e. Ziarat (r = 0.260), Khunjerab (r = 0.294), and Naltar (r = 0.258). Seasonal analysis of precipitation and runoff affirms that a significant correlation is lacking between summer (April to September) precipitation and summer runoff at Alam Bridge for the climate stations, as summarized in Table 4. A negative insignificant correlation is found between runoff at Alam Bridge and summer precipitation at Gilgit (r = −0.195), Ziarat (r = −0.101), and Naltar (r = −0.130). A significantly negative correlation is found at the Gupis (r = −0.238) and week positive correlation Khunjerab (r = 0.148) stations.

## 4. Discussion

MODIS snow cover for the study period indicates that the SCA in the Gilgit River Basin has a slightly decreasing trend, although the trend is not statistically significant. Decreasing snow cover and shrinking of glaciers provoke apprehensions about decreasing water in northern Pakistan [29]. However, Tahir et al. [4] affirmed slightly increasing trends in the maximum and minimum SCAs in Hunza Basin, whereas Farhan et al. [30] corroborated that a significant trend does not exist in the minimum and maximum SCA in the Astor Basin. This slightly decreasing trend in the cryosphere pattern might be the reason of climate variability because changes in climate not only affect the timing of winter snow fall but also its amount. Warm climate decrease snowfall, causes snow to melt earlier in spring, and reduces snow cover season. Seasonal average SCA (%) analysis shows that, spring season has the greatest SCA compared with winter season, which might mean more snowfall occurs in spring instead of winter. Snow accumulation at the end of winter does not have sufficient time to complete metamorphic process for conversion of snow into ice, melting immediately in early summer [31]. Seasonal SCA anomalies also shows a non-significant decreasing trend however, autumn season shows a statistically significant decreasing trend. According to the Intergovernmental Panel on Climate Change statement [32], seasonal snow cover duration in Chinese alpine areas is estimated to shorten and decrease in volume, which can lead to severe spring drought and similar magnitude of changes in other major river systems of China and Asia [29].

The UIB that mostly received annual precipitation in winter and spring originated from westerly [6], and monsoonal influences is negligible in summer; consequently, the climate pattern in UIB is different from that in eastern Himalayas [17]. The precipitation record of climate stations in Gilgit River Basin shows a non-significant increasing trend in all stations except for Gilgit and Gupis which shows a decreasing trend. These two stations are installed on the valley, where the precipitation pattern of the basin does not show the change with the altitude. The climatic variation in the Basin might be explained by the installation of climate stations at different altitudes of 1460–4730 m above sea level because the climate variables (especially precipitation) are strongly influenced by altitude [7]. In the high mountains of the Karakorum, the active hydrological region for the UIB lies at high altitude. According to the study conducted by Archer [16], the 36% catchment area of UIB at Partab Brigade above 5000 m is mainly fed by melting of snow and glaciers. Khunjerab climate station is situated at higher altitude just below 5000 m, representing better climatic pattern at the active hydrological zone of the Gilgit River Basin. This climate station record shows the increasing trend of temperature (annual and seasonal). According to the Shrestha et al. [33] study, HKH region is under the immense influence of global warming because it is warming more than the world average. By 2050, temperatures across the HKH region are projected to increase by nearly 1–2 °C on average [33]. Winter temperature also shows an increasing trend in the basin which may change the solid precipitation on to liquid. A significant increase in winter temperature in UIB leads to change in the amount of solid precipitation as a rainfall near the freezing point, thereby resulting in snow-fed catchment runoff being affected significantly compared with the glacier-fed catchments [17].

Snow dynamics has been accounted as an important variable in understanding, modeling, and predicting various atmospheric, ecological, and hydrological processes in snow-dominated basins [34]. Person correlation coefficient indicates that SCA has a highly significant negative correlation with annual temperature and stream flow in the Basin. A correlation coefficient of -0836 for summer stream flow indicates that Gilgit River runoff is highly dependent on SCA and mean temperature. The results are consistent with other studies in this region [17,30,35] The results of Tahir et al.’s [36] comparative study of the Hunza and Astor basins obtained a high correlation between SCA and mean temperature as well as for SCA and stream flow for the Hunza Basin. Significant positive correlation coefficient values of annual and seasonal temperature with runoff indicates that Gilgit River flow is driven by snow and ice melt. Archer D. [16] explained that the summer highest flows in Karakorum region results from the heat energy, melting the snow packs where water is stored in the form of ice and snow. The results corroborate that the Gilgit River flow is driven by snow and ice melt, in accordance with several investigations in which the westerly dominated river basins’ contribution of snow and glacial melt to river flow is more significant than monsoon-dominated precipitation regimes [2,37]. It has been observed that the mean temperature has a strong correlation with summer runoff in the feeding tributaries of UIB dominated by glacier and snow melt [17]. Forsythe et al. [38] speculated that a 1 °C increase in mean summer temperature may cause nearly a 10–20% increase in summer runoff into the Hunza river. Tahir et al. [4] also affirmed a strongly positive correlation between the average temperatures of the Astor and Hunza basins with their respective rivers.

Annual precipitation does not have a significant correlation with SCA and stream flow in Gilgit River Basin. This poor correlation may because several meteorological stations are installed at the valleys; thus, recorded data could not represent precipitation at a high elevation. Another reason for this weak correlation might be the resulting from various errors in instrumentation and recording [39]. The Khunjerab climate station (r = −0.124), which is below 5000 m, has the lowest precipitation record because the high-altitude climate station only catches 20–30% of precipitation while strong winds disperse the rest of the gauge [40,41]. Winiger et al. [20] projected a five to tenfold increase in precipitation for an elevation of more than 5000 m. By combining the snow cover and ablation model in HKH region, a large drop in temperature occurred. The reason for the negative correlation is the large proportion of annual precipitation which falls as solid precipitation, especially at high altitudes [33], and takes time to be part of river flow. Increased cloudiness during precipitation, which obscures insolation and intense reflection of short-wave radiation from fresh snow, is the factor responsible for snowmelt; thereby resulting in low stream flow [17]. This may also be because the high mountains of the Karakorum act as a barrier; hence, the monsoon effect over the UIB region is minimum [42].

## 5. Conclusions

MODIS remote sensing SCA and hydrometerological ground stations data were analyzed to study the spatio–temporal dynamics of snow cover and its relationship with climate change and stream flow. The SCA values exhibit a statistically non-significant decreasing trend except for the autumn season, which shows a significant decreasing trend for the Gilgit River Basin. Spring season has more SCA may be the reason of changing snowfall pattern. Variability in SCA indicates the climate change, which affect snow fall timing and its amount in the basin. Winter temperature shows an increasing trend which may change the solid precipitation in to liquid near the freezing point. Furthermore, Pearson correlation coefficient of annual and seasonal temperature shows that SCA has highly negative correlation with the summer temperature as a result increased in river runoff in the basin. River runoff is highly dependent on snow cover change, and finding the snow accumulation in winter is very important to predict the peak flow in summer for better management of water resources. Precipitation has a weak correlation with the SCA and stream flow in the basin. The present precipitation record of climate stations is not a true representation because most of the stations are installed at the valleys while the high-altitude stations has a systematic errors in precipitation gauges. Although, this is the first study to contribute to the analysis of snow cover in one of UIB’s most important river basin, where the impact of global warming would have significances in water balance and, therefore, impact on the sustainable management of water resources.

## Figures and Tables

**Table 1:** Pearson correlation coefficients for observed monthly temperature and precipitation among the climate observatories for the period 1995–2013.

Observatory	Gupis				Khunjerab				Ziarat				Naltar			
	Tmax *	Tmin **	Tmean ***	Prec. ****	Tmax	Tmin	Tmean	Prec.	Tmax	Tmin	Tmean	Prec.	Tmax	Tmin	Tmean	Prec.
Gilgit	**0.993**	**0.956**	**0.968**	**0.624**	**0.984**	**0.941**	**0.981**	**0.168**	**0.977**	**0.956**	**0.982**	**0.207**	**0.987**	**0.863**	**0.962**	**0.512**
Gupis	-	-	-	-	**0.984**	**0.953**	**0.966**	**0.139**	**0.977**	**0.977**	**0.970**	**0.256**	**0.986**	**0.902**	**0.951**	**0.465**
Khunjerab	-	-	-	-	-	-	-	-	**0.984**	**0.971**	**0.986**	**0.412**	**0.987**	**0.816**	**0.945**	**0.364**
Ziarat	-	-	-	-	-	-	-	-	-	-	-	-	**0.985**	**0.892**	**0.964**	**0.307**
**Coefficient values were determined by using Pearson’s correlation with 95% confidence interval at *p* = 0.05**																

Note: Bold ― *p* < 0.05, *: Tmax is the monthly maximum temperature; **: Tmin is the monthly minimum temperature; ***: Tmean is the monthly mean temperature; ****: Prec. is the total precipitation.

**Table 2:** Mann–Kendall statistics for the five observatories (1995–2013). M-K, and P are the Kendall’s tau, and the two-side p-value, respectively. (+ and − indicates positive trend and negative trend, ^+^, * indicates significant and non-significant respectively at confidence level α=0.05). T indicates the temperature.

Observatory	Climatic Variables	M-K	*P*	Sen’s Slope	Trend
Gilgit	Annual T (Jan.–Dec.)	0.27	0.13	0.02	[+, *]
	Summer T (Apr.–Sep.)	0.11	0.57	0.02	[+, *]
	Winter T (Oct.–Mar.)	0.29	0.09	0.05	[+, *]
	Precipitation (Jan.–Dec.)	−0.06	0.76	−1.9	[−, *]
Gupis	Annual T (Jan.–Dec.)	0.05	0.82	0.02	[+, *]
	Summer T (Apr.–Sep.)	0.12	0.52	0.04	[+, *]
	Winter T (Oct.–Mar.)	0.11	0.54	0.01	[+, *]
	Precipitation (Jan.–Dec.)	−0.19	0.29	−9.56	[−, *]
Khunjerab	Annual T (Jan.–Dec.)	0.24	0.17	0.05	[+, *]
	Summer T (Apr.–Sep.)	0.18	0.59	0.02	[+, *]
	Winter T (Oct.–Mar.)	0.27	0.13	0.17	[+, *]
	Precipitation (Jan.–Dec.)	0.12	0.49	2.75	[+, *]
Ziarat	Annual T (Jan.–Dec.)	0.15	0.41	0.03	[+, *]
	Summer T (Apr.–Sep.)	−0.08	0.65	−0.01	[−, * ]
	Winter T (Oct.–Mar.)	0.24	0.17	0.05	[+, *]
	Precipitation (Jan.–Dec.)	0.24	0.17	7.3	[+, *]
Naltar	Annual T (Jan.–Dec.)	−0.29	0.11	−0.06	[−, * ]
	Summer T (Apr.–Sep.)	−0.33	0.06	−0.09	[−, * ]
	Winter T (Oct.–Mar.)	−0.20	0.26	−0.08	[−, * ]
	Precipitation (Jan.–Dec.)	0.23	0.19	14.98	[+, *]

**Table 3:** Annual and seasonal correlation coefficients between snow cover area, Gilgit River basin (2001–2013); (**a**) monthly climate data (temperature and precipitation) in the Gilgit Basin climate stations (2001–2013), and (**b**) stream flow at Alam Bridge.

	Climate Data	Snow Cover Dynamics in Gilgit River Basin		
		Annual Correlation (Jan. to Dec.)	Summer Correlation (Apr. to Sep.)	Winter Correlation (Oct. to Mar.)
**a. Mean Temperature**				
	Gilgit	**−0.612**	**−0.834**	0.049 (0.66)
	Gupis	**−0.626**	**−0.822**	−0.025 (0.83)
	Khunjerab	**−0.662**	**−0.832**	−0.078 (0.49)
	Ziarat	**−0.683**	**−0.892**	−0.101 (0.38)
	Naltar	**−0.635**	**−0.783**	0.010 (0.93)
	Avg. of Gilgit Basin	**−0.652**	**−0.873**	−0.027 (0.81)
**Precipitation**				
	Gilgit	0.001(0.99)	**0.248**	0.179 (0.12)
	Gupis	0.093(0.25)	**0.252**	**0.233**
	Khunjerab	−0.124(0.122)	**−0.274**	0.072 (0.53)
	Ziarat	0.121(0.13)	0.079 (0.48)	0.205 (0.71)
	Naltar	0.060(0.38)	**0.246**	−0.047 (0.68)
	Avg. of Gilgit Basin	0.076(0.34)	**0.248**	0.119 (0.29)
**b. Stream flow**		**−0.806**	**−0.836**	**−0.415**
	Summer Stream flow	-	-	0.063 (0.57)
Coefficient values were determined by using Pearson’s correlation with 95% confidence interval at *p* = 0.05				

Note: Bold―*p* < 0.05, Values in braces indicate *p*-value.

**Table 4:** Annual and seasonal correlation coefficients between stream flow, Gilgit River Basin at Alam Bridge (2001–2013), and mean monthly climate data (temperature and precipitation) in the Gilgit Basin climate stations (2001–2013).

Observatory	Climate Variables		Stream Flow in Gilgit River at Alam Bridge		
			Annual Correlation (Jan. to Dec.)	Summer Correlation (Apr. to Sep.)	Winter Correlation (Oct. to Mar.)
Gilgit	Temperature	Minimum	**0.811**	**0.906**	**0.377**
		Maximum	**0.797**	**0.850**	**0.623**
		Mean	**0.815**	**0.914**	**0.554**
	Precipitation		0.132 (0.10)	−0.195 (0.08)	**−0.254**
Gupis	Temperature	Minimum	**0.810**	**0.890**	**0.482**
		Maximum	**0.800**	**0.845**	**0.653**
		Mean	**0.810**	**0.882**	**0.588**
	Precipitation		−0.009 (0.91)	**−0.238**	−0.161 (0.15)
Khunjerab	Temperature	Minimum	**0.837**	**0.830**	**0.666**
		Maximum	**0.820**	**0.890**	**0.581**
		Mean	**0.836**	**0.884**	**0.640**
	Precipitation		**0.174**	0.148 (0.19)	0.075 (0.52)
Ziarat	Temperature	Minimum	**0.845**	**0.913**	**0.614**
		Maximum	**0.840**	**0.899**	**0.583**
		Mean	**0.846**	**0.909**	**0.612**
	Precipitation		−0.062 (0.44)	−0.101 (0.37)	−0.054 (0.64)
Naltar	Temperature	Minimum	**0.685**	**0.529**	**0.283**
		Maximum	**0.836**	**0.882**	**0.611**
		Mean	**0.784**	**0.750**	**0.459**
	Precipitation		−0.020 (0.80)	−0.130 (0.25)	−0.092 (0.42)
Avg. Gilgit Basin	Temperature	Mean Temperature	**0.829**	**0.907**	**0.591**
	Precipitation	Total Precipitation	0.028 (0.073)	−0.207 (0.06)	−0.112 (0.32)
**Precipitation Winter** (Oct. to Mar.)			**Summer Correlation** (Apr. to Sep.)		
Gilgit			0.189 (0.09)		
Gupis			0.139 (0.22)		
Khunjerab			**0.294**		
Ziarat			**0.260**		
Naltar			**0.258**		
Avg. of Gilgit Basin			**0.362**		
Coefficient values were determined by using Pearson’s correlation with 95% confidence interval at *p* = 0.05					

Note: Bold―*p* < 0.05, Values in braces indicate *p*-value.
